# The clinical aspects of the acute facet syndrome: results from a structured discussion among European chiropractors

**DOI:** 10.1186/1746-1340-17-2

**Published:** 2009-02-05

**Authors:** Lise Hestbaek, Alice Kongsted, Tue Secher Jensen, Charlotte Leboeuf-Yde

**Affiliations:** 1 Nordic Institute of Chiropractic and Clinical Biomechanics, Forskerparken 10B, DK-5230 Odense M, Denmark; 2 The Back Research Centre, Lindevej 5, DK-5750 Ringe, Denmark

## Abstract

**Background:**

The term 'acute facet syndrome' is widely used and accepted amongst chiropractors, but poorly described in the literature, as most of the present literature relates to chronic facet joint pain. Therefore, research into the degree of consensus on the subject amongst a large group of chiropractic practitioners was seen to be a useful contribution.

**Methods:**

During the annual congress of The European Chiropractors Union (ECU) in 2008, the authors conducted a workshop involving volunteer chiropractors. Topics were decided upon in advance, and the participants were asked to form into groups of four or five. The groups were asked to reach consensus on several topics relating to a basic case of a forty-year old man, where an assumption was made that his pain originated from the facet joints. First, the participants were asked to agree on a maximum of three keywords on each of four topics relating to the presentation of pain: 1. location, 2. severity, 3. aggravating factors, and 4. relieving factors. Second, the groups were asked to agree on three orthopaedic and three chiropractic tests that would aid in diagnosing pain from the facet joints. Finally, they were asked to agree on the number, frequency and duration of chiropractic treatment.

**Results:**

Thirty-four chiropractors from nine European countries participated. They described the characteristics of an acute, uncomplicated facet syndrome as follows: local, ipsilateral pain, occasionally extending into the thigh with pain and decreased range of motion in extension and rotation both standing and sitting. They thought that the pain could be relieved by walking, lying with knees bent, using ice packs and taking non-steroidal anti-inflammatory drugs, and aggravated by prolonged standing or resting. They also stated that there would be no signs of neurologic involvement or antalgic posture and no aggravation of pain from sitting, flexion or coughing/sneezing.

**Conclusion:**

The chiropractors attending the workshop described the characteristics of an acute, uncomplicated lumbar facet syndrome in much the same way as chronic pain from the facet joints has been described in the literature. Furthermore, the acute, uncomplicated facet syndrome was considered to have an uncomplicated clinical course, responding quickly to spinal manipulative therapy.

## Background

The term 'facet joint' became common in the 1970s, when surgeons developed an interest in the small joints of the lumbar spine as a source of low back pain. The formal name for these joints is the zygapophyseal joints, as endorsed by The International Anatomical Nomenclature Committee [1]. They were suggested as a source of pain as early as 1911 [2] and the term 'facet syndrome' was introduced by Ghormley in 1936 [3]. However, due to the discovery of the lumbar disc as a source of low back pain, the facet joints did not receive much further attention until the 1970s. In 1976, Mooney and Robertson demonstrated that the facet joints could be a source of pain and that certain patients could be relieved from pain by anesthetizing these joints [4]. These findings were later reproduced [5,6] and thus confirmed the basis for the concept of 'facet syndrome', 'facet joint pain' or 'zygapophyseal joint pain'. The term 'facet syndrome' is really a contradiction in terms. A syndrome is characterized by a set of detectable characteristics, usually used when the pathophysiology has not yet been discovered [7]. In the case of 'facet syndrome', the source of pain is identified but the clinical presentation is poorly defined. Nevertheless, the term is widely used and a Medline search in July 2008 on 'facet syndrome' yielded 351 hits.

During the past three decades, there have been numerous studies of the frequency of facet joint pain in chronic low back pain patients. In these studies, various types of facet joint injections were used to determine whether the facet joints were the source of pain. These included injection of local anaesthetic into the joint itself or the nerves that innervate them, resulting in relief from pain if the pain originated from these joints (diagnostic blocks). Prevalence rates of facet joint pain among those patients with chronic low back pain vary widely in the literature, ranging from 5% to 90% [8] but there is a problem with a high false positive rate in many studies. Therefore, when confirmatory blocks are used, the prevalence rates are somewhat lower, ranging from 9% [9] to 45% [10]. As these studies investigated chronic low back pain, these prevalence rates indicate that the facet joints might be important contributors to the burden of chronic low back pain. However, there does not appear to be any studies describing the prevalence of facet joint pain in acute low back pain.

The etiology of pain from the facet joints has been investigated from several perspectives. Osteoarthrosis has been considered as a source of facet joint pain. Facet joint osteoarthrosis is very common in the general population; the frequency increases with age and the highest prevalence is at the L4-5 spinal level [11]. However, the presence of osteoarthrosis in the facet joints, as seen on plain radiography, does not seem to be associated with low back pain [11,12]. In contrast, facet joint oedema visualised by MRI correlated with back pain intensity in at least two studies [13,14]. A common explanation in chiropractic textbooks is that small meniscoids formed of synovial folds and continuous with the periarticular tissues become entrapped or extrapped and through a cascade of events lead to acute locked low back. This is described as being amenable to manipulative therapy [15,16]. Garges, White and Koestler offer an alternative or supplementary explanation of pain from the facet joints. They describe how inflammatory adhesions of the facet joints and their capsules may cause a painful reduction in motion [17].

The trapped meniscoid and inflammatory adhesion explanations have given rise to the theory that the 'facet syndrome' is a lesion which responds well to manipulative therapy. Cassidy and Kirkaldy-Willis write: "An adjustment (manipulation) that separates the articular surfaces may release entrapped synovial folds and stretch the segmental muscles initiating spindle mediated reflexes that relieve the state of hypertonicity [of paraspinal muscles splinting the posterior joints]" [15], and Murphy et al postulate that the facet joints are the target of all successful spinal manipulation [17]. Likewise, Cox describes the facet syndrome as "probably the most common factor seen in chiropractic practices with low back pain patients...." [18].

It is therefore not surprising that the facet syndrome has a prominent place in chiropractic education and practice. Unfortunately, this is not reflected in research, which creates a gap between practice and scientific evidence. When practising evidence-based medicine, one has to draw on empirical evidence in the areas where scientific evidence is lacking. In the case of the acute facet syndrome, the amount of scientific evidence relating to diagnosis and treatment is almost non-existent. Despite this uncertainty, 'acute facet syndrome' appears to be a commonly used diagnosis in primary care among general practitioners, chiropractors and physiotherapists, at least in Denmark. Since the term 'acute facet syndrome' is widely used and accepted among chiropractors, research into the degree of consensus on the subject amongst a large group of practitioners was seen to be a useful contribution. Therefore, this study aims to describe chiropractors' views of the clinical presentation of, and course of treatment for, acute facet syndrome in the lumbar spine.

## Methods

During the annual congress of The European Chiropractors Union (ECU) in 2008, the authors conducted a workshop involving volunteer chiropractors from several European countries. Topics were decided upon in advance, and the participants were asked to form into groups of four or five. Between sessions, the participant mix in the groups was changed to avoid dominance by any individual.

The workshop was structured around a basic case, which involved a 40-year old man presenting with pain in the lower lumbar area of two days' duration. There were no additional musculoskeletal problems, no other health problems, no abnormal x-ray findings and no red flags. He appeared to be in good health, both physically and mentally, and there were no social or work-related problems. This case was chosen to make the clinical picture of 'acute facet syndrome' as clear and uncomplicated as possible.

The groups were asked to reach consensus on several topics relating to this case, where an assumption was made that the subject's pain originated from the facet joints. The workshop was divided into two sessions. In the first session, the participants were asked to agree on a maximum of three keywords on each of four topics relating to the presentation of pain: 1. location, 2. severity, 3. aggravating factors, and 4. relieving factors. The choice of these keywords was left entirely to the groups. The authors had not prepared a list of words to choose from nor provided any other type of guidance for the groups. In the second session, the groups were asked to agree on three orthopaedic and three chiropractic tests that would aid in diagnosing pain from the facet joints. It was explained that orthopaedic tests were clinical tests, expected to be used by all clinicians examining back pain patients, and chiropractic tests were tests believed to be used primarily by chiropractors. Again, the choice of tests in the two categories was left entirely to the groups. Then they were asked to reach agreement within their group regarding number, frequency and duration of chiropractic treatment.

When all groups had reached consensus on each topic, the results were written on flip charts by a group member. In the case of key words, these were grouped by the authors to identify agreement. For example, for the word "palpation", it was determined whether it meant motion or static palpation, and the total number of groups choosing static palpation and the total number of groups choosing motion palpation were summed. Where misunderstandings could occur due to the use of vague or differing terminology, group representatives were asked to explain further or to demonstrate the position or test in question. At the end of each session, there was a general discussion to identify and correct any misunderstandings. The data recorded on flip charts were copied by one of the authors and used to summarize the results.

After the first session, it was clear to the research team that many of the keywords could relate to acute low back pain in general. Therefore, another open discussion was conducted in the whole group, to identify features that were thought to distinguish acute low back pain arising in the facet joints from acute low back pain arising in the discs. A summary of this discussion is presented in this article, but results are not quantified.

## Results

In the first session, there were 24 male and 10 female participants; and 24 males and 8 females participated in the second session. The participants formed 7 groups during the first session and 8 groups during the second session. Chiropractors from Belgium, Denmark, France, Great Britain, Iceland, Italy, Norway, Sweden, and The Netherlands were present. Groups were formed irrespective of nationality.

### Summary of findings

#### Session 1: The characteristics of pain originating from the facet joints

Group conclusions with regard to the characteristics of pain originating from the facet joints are provided in Table 1 and described below.

**Table 1:** Typical presentation of an acute lumbar facet syndrome according to the 34 chiropractors participating in the structured group discussion.

**Location**	
Local	7 (100%)

Ipsilateral	7 (100%)

Possible referred pain no further than the knee	4 (57%)

Sclerogenic	1 (14%)

	
**Severity**	

Severe	3 (43%)

Moderate	2 (29%)

Variable (both between and within patients)	1 (14%)

Mild to severe	1 (14%)

Full range (mild to excruciating)	1 (14%)

	
**Aggravating factors**	

Extension	4 (57%)

Prolonged standing	3 (43%)

Rotation	3 (43%)

Sudden movements	2 (29%)

Worse after rest	2 (29%)

Ipsilateral lateral flexion	1 (14%)

Getting up from flexion	1 (14%)

Movement in general	1 (14%)

	
**Relieving factors**	

Walking	5 (71%)

Lying with knees bent (supine or on the side)	4 (57%)

NSAID	3 (43%)

Supported flexion, (resting on hands or elbows)	3 (43%)

Ice	2 (29%)

Short rest	2 (29%)

Rest	1 (14%)

Avoid aggravating factors	1 (14%)

Contralateral lateral flexion	1 (14%)

Varying activities for brief periods	1 (14%)

Keywords noted by the groups. Reported as number and percentages of the seven groups.

##### Location

All groups agreed that the pain would be local and ipsilateral (to the side of the facet joint involvement). Four groups (57%) considered referred pain and they all agreed that the pain could refer no further than the knee. One of these groups (14%) considered not only the location but also the type of pain believing it to be sclerogenic in nature.

##### Severity

There were rather different opinions about the severity of the pain, but no groups considered it to be consistently mild. Three groups (43%) believed that the pain would be severe and one group (14%) that it would be moderate in intensity. The remaining three groups (43%) thought that the pain intensity could be anything from mild to severe. One of these groups (14%) also mentioned that the pain could vary not only from individual to individual but also within the same individual.

##### Aggravating factors

The most commonly agreed aggravating factor was extension (57%) followed by rotation and prolonged standing (43%). Two groups (29%) expected the pain to worsen with sudden movements and after resting. Furthermore, lateral flexion towards the involved side, returning from a flexed position, and movement in general was mentioned.

##### Relieving factors

A majority of the groups (71%) assumed the pain would lessen with walking or lying with knees bent (57%). Three groups (43%) believed that non-steroidal anti-inflammatory drugs (NSAIDs) would relieve the pain, and two groups (29%) that cold packs would do likewise. Three groups (43%) thought pain could be relieved by supported flexion, sitting or standing, with the weight resting on hands or elbows. Short rest was mentioned by two groups (29%). Rest (in general), avoidance of aggravating factors, lateral bending away from the pain and varying activities for brief periods were all mentioned by one group (14%).

Although when asked to agree on a maximum of three words for each subject the groups mentioned different factors, none of the chosen words seemed to contradict each other. Also, when comparing the list of aggravating factors with the list of relieving factors, they seemed obvious opposites, for example, prolonged standing was considered aggravating and walking was considered relieving.

#### Session 2: Examination findings and management of pain originating from the facet joints

Group conclusions with regard to the examination findings for pain originating from the facet joints are provided in Table 2 and described below.

**Table 2:** Typical clinical findings of an acute lumbar facet syndrome according to 32 European chiropractors participating in the structured group discussion.

**Orthopaedic examination**	
Kemp's test (sitting rotation and extension)	7 (88%)

Pain and/or decreased extension	4 (50%)

Pain and/or decreased extension + lateral flexion	3 (38%)

No neurological involvement	3 (38%)

Springing test (prone segmental extension)	2 (25%)

Relief in supine flexion with knees bent	1 (13%)

Painful end range of motion in all directions	1 (13%)

Pain on prone active extension	1 (13%)

Yeoman's test modified for lumbar segmental extension	1 (13%)

Decreased contralateral rotation	1 (13%)

Palpatory tenderness	1 (13%)

	
**Chiropractic examination**	

Static palpation (pain)	8 (100%)

Motion palpation (decreased motion)	8 (100%)

Local muscle spasm	2 (25%)

Motion palpation (pain)	2 (25%)

Oedema	1 (13%)

Antalgia	1 (13%)

Springing test	1 (13%)

Applied Kinesiology challenge	1 (13%)

Break in curvature on lateral flexion	1 (13%)

Keywords noted by the groups. Reported as number and percentages of the eight groups.

##### Orthopaedic examination

When discussing positive orthopaedic tests, all groups considered extension to be painful and/or decreased. Three of these groups (38%) combined extension with lateral flexion and one group (13%) specified the extension as active and prone. Almost all groups (88%) expected Kemp's test to be positive and three groups mentioned the absence of neurological signs. When combining orthopaedic and chiropractic tests, the springing test was thought to be positive by three groups (38%), although one group mentioned this under 'chiropractic tests '.

##### Chiropractic examination

In relation to the specific chiropractic examination, everybody agreed that there would be pain on static palpation and decreased motion as detected on motion palpation. Two groups (25%) also expected pain on motion palpation and two groups (25%) anticipated local muscle spasm. Furthermore, oedema, antalgia, springing test (see orthopaedic tests), applied kinesiology challenge and break in curvature in lateral flexion were mentioned.

Group conclusions with regard to the clinical course of treatment of pain originating from the facet joints are provided in Table 3 and interpreted below.

**Table 3:** Typical course of treatment of an acute lumbar facet syndrome according to 32 European chiropractors, divided into 8 groups and participating in the structured group discussion.

**Number of treatments needed**		**Number of groups, n (%)**
1 – 4		1 (13%)

2 – 3		1 (13%)

2 – 4		1 (13%)

2 – 5		1 (13%)

4		1 (13%)

3 – 5		1 (13%)

3 – 6		1 (13%)

3 – 9		1 (13%)

**Duration of treatments, weeks**		**Number of groups, n (%)**

2		2 (25%)

2 – 3		2 (25%)

2 – 4		2 (25%)

1 1/2 – 2		1 (13%)

4 – 5		1 (13%)

		
**Number of treatments per week**		

**1^st ^week**	**2^nd ^week**	**3^rd ^week**

1–2*	2*	0*

2	1 – 2	0 – 1

2	0 – 2	0

2 – 3	1 – 2	1

2 – 3	1 – 2	0

2	1 – 2	0 – 1

1 – 2	1	0 – 1

* This sequence of treatment numbers seems illogical. There might be a mistake in recording.

##### Number of treatments needed

With regard to the number of treatments in a course of treatment, one group wrote four, but the other seven groups all stated a range. This ranged from 1–4 to 3–9 treatments, that is, all groups included three treatments.

##### Duration of treatment

With the exception of one group, the beliefs were fairly uniform, close to two weeks, in relation to the duration of treatment. The shortest period of treatment proposed was from one and a half to two weeks and the longest period was 4–5 weeks. All, but the group proposing 4–5 weeks, had two weeks in the range of their answer.

##### Number of treatments per week

Number of treatments ranged from one to three the first week, none to two the second week and none to one the third week – all showing a fairly uniform pattern of two treatments in the first week, one treatment in the second week and no treatments in the third week.

##### Open discussion to highlight factors which distinguish pain originating from the facet joint from pain originating from the discs

During the discussion there was general agreement on the following. Signs and symptoms which the chiropractors believed to be specific for discogenic pain and **not **found in an acute facet syndrome were: antalgia/lateral shift, limping, parasthesia, and radicular leg pain. Aggravating factors which the chiropractors believed to be specific for discogenic pain and **not **found in an acute facet syndrome were: sitting, flexion, using a clutch (in a vehicle), coughing and/or sneezing, and walking for a long time.

One group mentioned antalgia as a sign of facet joint pain in the group discussions. Thus, there was some disagreement with the conclusion from the general discussion that antalgia was specific for discogenic pain. No other disagreement was noted.

##### Analytical problem

When analyzing these data, it became apparent that the summary report from the workshop contained an unexplained irregularity. The number of keywords in one of the topics (orthopedic tests) added up to more than the number of groups (eight) multiplied by the keywords (three). That is, the orthopedic tests summed to 25 where it should have summed to 24. One explanation could be that a group used a single word which was later interpreted in the analysis as covering two concepts, such as 'palpation' which was interpreted as both motion and static palpation. However, at the time of writing, the original data recorded on flip charts were no longer available, so the discrepancies in numbers could not be explained.

## Discussion

Generally, the participating chiropractors' views of the acute facet syndrome and the description of chronic facet joint pain found in the existing literature were surprisingly similar. The chiropractors attending the ECU workshop described the characteristics of an acute, uncomplicated facet syndrome as follows: local, ipsilateral pain, occasionally extending into the thigh with pain and decreased range of motion in extension and rotation both standing and sitting. They thought that the pain could be relieved by walking, lying with knees bent, using ice packs and taking NSAIDs, and aggravated by prolonged standing or resting. They also stated that there would be no signs of neurologic involvement and no sign of aggravation of pain from sitting, flexion or coughing/sneezing. Finally, they did not link the acute facet syndrome with an antalgic posture. These findings have been summarized in Table 4 which also includes findings from the literature.

**Table 4:** Overview of findings from the literature combined with the findings from the workshop described in the present study.

**Location of pain in patients with lumbar facet syndrome**					
*Study*	*local*	*paraspinal*	*thigh/groin*	*calf/foot*	

Fairbank 1981	-	-	pos.	neg.	

Lippitt 1984	pos.	pos.	pos.	-	

Lewinneck 1986	-	pos.	-	-	

Helbig 1988	-	pos.	pos.	neg.	

Results from present study	pos.	-	pos.	neg.	

					
**Painful movements in patients with lumbar facet syndrome**					

*Study*	*flexion*	*extension/hyper-extension*	*rotation*	*rising from flexion*	*rising from sitting*

Fairbank 1981	pos.	-	-	-	-

Lippitt 1984	-	pos.	-	-	-

Helbig 1986	-	pos.	pos.	-	-

Revel 1992	neg.	neg.	neg.	neg.	-

Markwalder 1994	neg.	pos.	-	-	-

Schwarzer 1995	no	no	no	-	-

Revel 1998	neg.	neg.	neg.	neg.	-

Manchikanti 2000	no	no	no	-	-

Young 2003	-	-	-	-	pos.

Laslett 2004	-	-	-	neg.	-

Laslett 2006	-	pos.	pos.	-	-

Results from present study	-	pos.	pos.	-	-

					
**Signs of nerve root compression in patients with lumbar facet syndrome**					

*Study*	*'No sign of nerve root compression/tension'*	*No aggravation with straight leg raising*	*No pain coughing*		

Lippitt 1984	pos.	-	-		

Lewinneck 1986	pos.	-	-		

Helbig 1988	neg.	-	-		

Jackson1988	pos.	-	-		

Schwarzer 1995	-	no	-		

Revel 1992	-	-	pos.		

Revel 1998	-	-	pos.		

Manchikanti 1999	-	pos.	-		

Manchikanti 2000	-	pos.	-		

Manchikanti 2000	-	pos.	no		

Laslett 2004	-	-	pos.		

Results from present study	pos.	-	-		

					
**Pain relieving positions in patients with lumbar facet syndrome**					

*Study*	*Sitting*	*Supine/recumbent*	*Walking*		

Revel 1992	-	pos.	-		

Revel 1998	-	pos.	-		

Manchikanti 2000	-	pos.	-		

Laslett 2006	pos.	-	pos.		

Results from present study	-	pos.	pos.		

pos: positive association

neg.: negative association

no: no association

-: not investigated

When interpreting results from the detailed discussion in Tables 1, 2 and 3, it must be remembered that the groups were asked to agree on a maximum of three words. Thus, even if a term is only mentioned by one group, this does not necessarily mean that the other groups disagree. For example, to the question of relieving factors, one group chose to answer "avoid aggravating factors". Although this was only mentioned by one group, it is likely that the other groups would agree. The authors decided they could only focus on obvious sources of disagreement in cases where the groups clearly contradicted each other and this did not happen in the study.

Since the early 1980s, several investigators have attempted to define clinical criteria to distinguish pain from the facet joints from other types of low back pain, but some results have been contradictory. Several authors agree that absence of positive signs of neurological compromise, such as positive straight leg raising, pain on coughing and dermatomal radicular pain, increases the likelihood of the pain originating in the facet joints [10,19-25]. Likewise, there is some agreement that the frequency of facet joint involvement in chronic low back pain increases with age [24-28], but Manchikanti did not find an association with age in two earlier studies [10,19]. Other studies failed to find any associations between response to facet joint blocks and patient history or physical examination, including straight leg raising and pain on movement [29,30]. With the exception of Fairbank et al's study from 1981[5], all of these studies included only patients with chronic low back pain. The essence is that so far only diagnostic blocks (including confirmatory blocks), not clinical signs and symptoms, can accurately diagnose back pain arising from the facet joints, regardless of whether it is acute or chronic.

Regarding the distribution of facet joint pain, some investigators have found it to be paraspinal [22,23,31], three studies have found pain extending into the groin or thigh [5,22,31] and two found pain extending into the calf to be a negative indicator for facet joint pain [5,31]. If the pain does extend into the leg it seems to be in the sclerodermal structures, referred from the nociceptors of the facet joint capsules [32].

The chiropractors in the current study agreed with the existing literature on chronic low back pain: local, paraspinal pain in the back, occasionally referring to groin and thigh, rarely below the knee. There was no information to be extracted from the workshop with regard to the intensity of pain, and interestingly, literature on this subject has not been found either. One finding that was a surprise was the chiropractors' belief that the pain is located to the side of involvement. Mention of such laterality in the literature has not been found, nevertheless, all groups agreed on the pain only being present on the side of involvement.

As for aggravating and relieving types of movement, there is more disagreement in the existing literature. Fairbank reported pain on flexion, whereas absence of pain aggravation on forward flexion was reported in three other studies [21,25,32]. Revel also reported absence of pain aggravation with extension and rotation [21,25] while increased pain on extension was found in four other studies [22,26,31,32] and increased pain on rotation in two [26,31]. Furthermore, pain relief from lying supine/recumbent [19,21,25], from walking [26] and from sitting [26] has been reported. Finally, absence of pain when rising from sitting [33] or flexion [20,21] has been shown to distinguish facet joint pain from pain from other structures.

Also for aggravating and relieving types of movement, the participants in this study agreed to a large extent with the literature pertaining to chronic facet syndrome: primarily pain on extension and rotation and relief from walking and lying down. In addition, the chiropractors also considered sudden movements, prolonged standing and prolonged rest as aggravating factors. The authors have not found any evidence for or against this in the literature. The same was true for supported flexion, icepacks and NSAIDs as relieving factors. This might be because the retrieved literature related to the distinction between pain originating from the facet joints and pain from other structures. Supported flexion, icepacks and NSAIDs might be relevant for all types of acute low back pain. The only clear discrepancy between the responses in this study and the existing literature is that one of the study groups considered getting up from flexion an aggravating factor whereas both Revel [21,25] and Laslett [20] found that absence of aggravation of pain on rising from flexion was characteristic of pain originating from the facet joints.

The results of the open discussion with all participants on how to distinguish the acute pain of facet joints from that of discal structures was also in concordance with the existing literature relating to chronic pain. It was believed that pain on sitting, flexion and prolonged walking would indicate disc rather than facet joint involvement and the same symptoms would indicate a typical radicular pattern, especially if accompanied by coughing and sneezing. One thing raised in the discussion, which is not mentioned in the facet joint literature, was antalgic posture as a sign of discogenic pain but not a sign of facet joint pain. This might be because the literature primarily considered chronic pain, in which antalgia is less common.

The participants in this study generally considered the syndrome to have an uncomplicated course, typically requiring 2–4 treatments over a period of two weeks. There did not appear to be any existing literature with regard to chiropractic management of the acute facet syndrome.

The high agreement among chiropractors and between chiropractors and the literature may indicate that chiropractors have a common educational background, and that their beliefs about facet joint pain to a large degree reflect what they were taught. Since chiropractors have a profound belief that facet joint pain responds well to manipulation therapy, it is likely that this description is actually of the typical patient responding well to manipulation rather than a patient with acute pain originating in the facet joints. Nevertheless, it is possible that these results do indeed capture the clinical picture of a patient with facet joint pain.

Based on the opinion of the chiropractors in this study, two hypotheses can be generated: (1) acute facet joint pain can be clinically defined, and (2) acute facet joint pain responds well to spinal manipulative therapy. Both of these hypotheses can be tested by verifying diagnoses using diagnostic blocks. The results would have implications for clinical decisions with regard to diagnosis and treatment for patients with low back pain. Since the clinical presentation thought to represent this condition is rather common and, to date poorly investigated, it is hoped that the above hypotheses will be tested in the near future.

## Conclusion

The chiropractors attending the workshop seemed to have a common understanding of pain originating from the facet joints. They described the characteristics of an acute, uncomplicated lumbar facet syndrome in much the same way as chronic pain from the facet joints has been described in the literature.

Furthermore, the acute, uncomplicated facet syndrome was considered to have an uncomplicated clinical course, responding quickly to manipulative therapy – a concept that appears never to have been scientifically tested.

## Competing interests

The authors declare that they have no competing interests.

## Authors' contributions

All authors planned and conducted the workshop. AK summarized the workshop. LH drafted the manuscript. All authors read and approved the final manuscript.
